# Post-diagnostic statin use and breast cancer-specific mortality: a population-based cohort study

**DOI:** 10.1007/s10549-022-06815-w

**Published:** 2023-03-17

**Authors:** Oliver William Scott, Sandar TinTin, Sixten Harborg, Marion J. J. Kuper-Hommel, Ross Lawrenson, J. Mark Elwood

**Affiliations:** 1 Department of Epidemiology and Biostatistics, School of Population Health, University of Auckland, Building 507, 85 Park Road, Grafton, Auckland, 1023 New Zealand; 2 Department of Oncology, Aarhus University Hospital/Aarhus University, Entrance C, Level 1, C118, Palle Juul-Jensens Boulevard 99, 8200 Aarhus N, Denmark; 3 Department of Oncology, Waikato District Health Board, Hamilton, New Zealand; 4 Department of NIDEA (National Institute of Demographic and Economic Analysis), Waikato Medical Research Centre, University of Waikato, Hamilton, New Zealand

**Keywords:** Breast cancer, Mortality, Statins, Pharmacoepidemiology, Cohort study

## Abstract

**Purpose:**

Statins are the most widely prescribed cholesterol lowering medications and have been associated with both improved and unchanged breast cancer outcomes in previous studies. This study examines the association between the post-diagnostic use of statins and breast cancer outcomes (death and recurrence) in a large, representative sample of New Zealand (NZ) women with breast cancer.

**Methods:**

Women diagnosed with a first primary breast cancer between 2007 and 2016 were identified from four population-based regional NZ breast cancer registries and linked to national pharmaceutical data, hospital discharges, and death records. Cox proportional hazard models were used to estimate the hazard of breast cancer-specific death (BCD) associated with any post-diagnostic statin use.

**Results:**

Of the 14,976 women included in analyses, 27% used a statin after diagnosis and the median follow up time was 4.51 years. Statin use (vs non-use) was associated with a statistically significant decreased risk of BCD (adjusted hazard ratio: 0.74; 0.63–0.86). The association was attenuated when considering a subgroup of ‘new’ statin users (HR: 0.91; 0.69–1.19), however other analyses revealed that the protective effect of statins was more pronounced in estrogen receptor positive patients (HR: 0.77; 0.63–0.94), postmenopausal women (HR: 0.74; 0.63–0.88), and in women with advanced stage disease (HR: 0.65; 0.49–0.84).

**Conclusion:**

In this study, statin use was associated with a statistically significant decreased risk of breast cancer death, with subgroup analyses revealing a more protective effect in ER+ patients, postmenopausal women, and in women with advanced stage disease. Further research is warranted to determine if these associations are replicated in other clinical settings.

**Supplementary Information:**

The online version contains supplementary material available at 10.1007/s10549-022-06815-w.

## Introduction

Breast cancer is the most common cancer in women and the leading cause of female cancer mortality worldwide [1]. Comorbidities are common in patients with breast cancer [2], and there is a high and increasing prevalence of risk factors for both breast cancer and ischemic heart disease among Western women [3–5]. As such, many patients with breast cancer use prescribed medications for cardiovascular conditions. Examining the association between commonly used cardiovascular medications and breast cancer outcomes is therefore warranted. Statins (3-hydroxy-3-methylglutaryl coenzyme A reductase inhibitors) are the most widely prescribed cholesterol lowering medications [6] and are used for both the primary and secondary prevention of cardiovascular disease [7].

Statins reduce cholesterol levels by inhibiting the rate limiting enzyme of the mevalonate pathway (HMGCR), which has been shown to be over expressed in breast cancer tumours [8, 9]. Statins have been found to exert pleiotropic effects, such as the induction of apoptosis, inhibition of proliferation, as well as expressing immunomodulatory properties [10–12]. In preclinical studies, statins have also reported to be associated with anti-neoplastic properties in animal models and breast cancer cell lines [13–15]. Several observational studies have been carried out on the basis of this evidence, and a number have reported a protective association between statin use and breast cancer-specific death (BCD) [16–22]. However, there are also some studies that have reported a null association between statin use and BCD [23–32]. Similarly, a number of observational studies have found a protective association between statin use and breast cancer recurrence (BCR) [27, 31, 33–36], while some have reported a null association [29, 30, 32, 37–42]. Two presurgical clinical trials in breast cancer patients have indicated that short-term fluvastatin and atorvastatin use may indeed exhibit antiproliferative activity (as measured by the Ki67 index) and increased apoptosis in high grade and HMGCR expressing tumours [43, 44]. Further, retrospective analysis of a phase three clinical trial (BIG 1–98) found that concurrent use of cholesterol lowering medication (CLM) with endocrine treatment was associated with improved disease-free survival in a large cohort of postmenopausal breast cancer patients [45].

There is conflicting evidence on the potential protective effect of statins with respect to the molecular subtype of breast cancer. Some studies indicate that statins exhibit a more protective effect in ER+ tumours [16, 17, 21, 33, 45–47], while some suggest that they may exert a more protective effect in ER- tumours [15, 48] (including triple negative breast cancer [20, 35, 49–58]). There has also recently been conjecture in the literature that the effect of statins may differ depending on the site of recurrence, and it is not clear if statins are more protective for local or distant recurrences [33, 36]. Therefore, our primary objective was to address this discordant evidence and explore the relationship between any post-diagnostic statin use and BCD in a large population-based cohort study of newly diagnosed patients with breast cancer in New Zealand. A secondary objective was to further elucidate the association between any post-diagnostic statin use and breast cancer recurrence.

## Methods

### Data sources

Eligible women were all those with a first primary breast cancer diagnosed and recorded in any of four population-based regional breast cancer registries (Auckland, Waikato, Wellington, and Christchurch) [59] in New Zealand between 1 Jan 2007 and 31 Dec 2016. These registers include all women diagnosed with breast cancer in their defined areas, and together cover about 70% of all breast cancer registrations in New Zealand. Using an anonymised National Health Index number, data were linked to several national data bases: the Pharmaceutical Collection (PHARMS), a national database containing dispensing information and medication identifiers from pharmacists for subsidised dispensings (from medical prescriptions exclusively; over the counter sales are not included) [60]; the National Minimum Dataset, relating to all day patients and inpatients discharged from both public and private hospitals; and the National Mortality Collection, with information about all certified deaths [61]. Women were excluded if their records did not link to at least one dispensing from the pharmaceutical collection (*n* = 14) or if their date of death was on or before their recorded date of breast cancer diagnosis (*n* = 3). The final cohort for analyses was comprised of 14,976 women.

### Exposure and outcome data

In the PHARMS database, medications dispensed any time after breast cancer diagnosis were determined using the therapeutic group ID, a PHARMAC identifier for each group of Anatomical, Therapeutic, and Chemical properties [60]. All statins dispensed to women in our cohort (atorvastatin, pravastatin, and simvastatin) were included. For each dispensing, we calculated the number of daily defined doses by multiplying the number of tablets dispensed by the dose per tablet in mg, and dividing by the daily defined dose in mg from the World Health Organisation database [62].

Deaths were determined from the underlying cause of death in the regional breast cancer registries and National Mortality Collection, with ICD codes C50.0 to C50.9 classified as deaths from breast cancer.

### Confounders

Demographic and clinical information came from the regional breast cancer registries, and covariates considered included date of diagnosis, age, ethnic group [63, 64], socioeconomic deprivation (NZDep) [65], urban/rural status [66], public/private status of the treatment facility, register, stage [67], grade [68], mode of detection (screen detected vs symptomatic), lymphovascular invasion, and molecular subtype (as defined previously [69], including Luminal A, Luminal B, Luminal B HER2+, HER2+ non-luminal, and triple negative). Other post-diagnostic medications included beta blockers, angiotensin converting enzyme inhibitors (ACEIs), angiotensin receptor blockers (ARBs), diuretics, metformin, tamoxifen, and aromatase inhibitors. Comorbidities adjusted for included any cardiac condition (angina, arrhythmia, congestive heart failure, hypertension, myocardial infarction, ‘other cardiac conditions’, and valve disease) as yes/no, diabetes, stroke, chronic obstructive pulmonary disorder, peripheral vascular disease, and renal disease. We defined comorbidities as any of the above conditions appearing in a patient’s linked hospital record (inpatient admissions) in the 5-year period before their breast cancer diagnosis. Cancer treatments such as chemotherapy and radiotherapy were not considered as confounders because these variables are highly correlated with covariates already adjusted for (such as stage and grade).

### Statistical analyses

Comparisons by statin use at baseline (date of diagnosis of breast cancer) were conducted using the chi-square test. We used Cox proportional hazard models to assess hazard ratios (HRs) of breast cancer-specific mortality associated with any post-diagnostic statin use vs non-use. Death registrations and Pharmaceutical Collection coverage were complete to the end of 2017, so we followed patients from their breast cancer diagnosis until death or 31 December 2017. Women with no death recorded prior to 31 December 2017 were assumed to be alive as at 31 December 2017. Medication use was conceptualised as a time varying covariate (with all women considered nonusers at baseline), such that time before the first dispensing was counted as ‘nonuser’ time, and time from the first dispensing to end of follow up was counted as ‘user’ time [70]. Models were adjusted in a systematic fashion, with the first adjustment including demographic and breast cancer clinical data, and the second adding other medication use and comorbidities.

Analyses were conducted considering statin use as a binary variable (user/nonuser), and also by splitting statin use into seven categories based on the number of daily defined doses (DDDs: categorised as 1-90 DDDs, 91-181 DDDs, 182-272 DDDs, 273-364 DDDs, 365-729 DDDs, 730-1094 DDDs, or 1095 or more DDDs, corresponding to the equivalent of 0–3 months, 3–6 months, 6–9 months, 9 months-1 year, 1–2 years, 2–3 years, and 3+ years of statin use respectively). Dose analyses were conducted using a time varying approach, such that women spent time in the lowest category before moving into the next dose category.

As dispensings towards the end of life may reflect changes in morbidity (including cancer recurrence/progression) or in health care related to end of life care [71, 72], we also conducted analyses lagging medication times [73]. In these analyses, patients are initially considered nonusers and then users after a lag period has elapsed after their first medication dispensing. Using this approach, dispensings towards the end of life are removed by the lag; for example, a 6-month lag will ignore dispensings in the 6 months prior to death/last follow up and classify these women as medication nonusers as opposed to users. To appropriately account for different periods in which end of life care may be administered, we also considered lag periods of 1 year and 2 years. In these analyses, all medications were modelled in the same fashion (for example, if statins were lagged by 6-months, all other medications were as well).

To evaluate the effect of the competing risk of death from other causes, the proportional subhazards model was also used [74]. For this analysis, all deaths apart from breast cancer deaths were treated as competing events.

We stratified the main analysis by estrogen receptor (ER) status to explore the relationship between statin use and tumours expressing different ER profiles (ER+ vs ER−). We also stratified the main analysis by triple negative status (cancers that were ER-, PR-, and HER2- vs cancers not fulfilling these criteria). In all of these analyses, patients with an ‘unknown’ ER, PR, or HER2 status were excluded where appropriate.

To investigate the effect of statin use in a more homogenous group and one in which the mechanisms of estrogen production [75] (and therefore cholesterol levels [76]) may differ, another analysis was conducted restricting the cohort to postmenopausal women only.

To examine the effect of statin use in early-stage patients only, an analysis was carried out restricted to patients with stage 1, stage 2, and stage 3a cancers. An analysis was also carried out in late-stage (stage 3b, stage 3b, and stage 4 cancers) patients. In these analyses, patients with an ‘unknown’ stage were excluded. 

In order to address the selection bias inherent in analysing both incident and prevalent users together [77–80], an analysis was carried out splitting these users into different categories. Incident/new statin users were defined as women who did not have a statin dispensing in the year prior to breast cancer diagnosis, while prevalent users were defined as those who did have a statin dispensing in the year prior to breast cancer diagnosis.

In order to compare statin users to patients using a different medication for a similar indication, a further analysis was carried out comparing statin users to statin nonusers who were dispensed aspirin. In this analysis, statin nonusers who used aspirin were followed from their first post-diagnostic aspirin dispensing until death or 31 December 2017.

We also conducted an analysis with breast cancer recurrence (BCR) as the outcome. In this analysis, we defined a BCR as either a local/regional recurrence or distant metastasis and restricted the cohort to patients with early-stage breast cancer as above. Recurrences were determined from the breast cancer registry data through patient’s routine clinical records, and women were followed from their breast cancer diagnosis until BCR, death, last follow up date, or end of Pharmaceutical Collection coverage (31 December 2017), whichever came first. These analyses examined risk of BCR associated with statin use vs non-use, and the same analyses were also carried out separately for local and distant recurrences.

Finally, we also conducted an analysis with all-cause mortality as the outcome. In this analysis, women were followed from their breast cancer diagnosis until death or 31 December 2017. Women with no death recorded prior to 31 December 2017 were assumed to be alive as at 31 December 2017.

Comparisons by statin use at baseline (date of diagnosis of breast cancer) were conducted using the chi-square test. We used Cox proportional hazard models to assess hazard ratios (HRs) of breast cancer-specific mortality associated with any post-diagnostic statin use vs non-use. Death registrations and Pharmaceutical Collection coverage were complete to the end of 2017, so we followed patients from their breast cancer diagnosis until death or 31 December 2017. Women with no death recorded prior to 31 December 2017 were assumed to be alive as at 31 December 2017.

Results are reported as HRs and their 95% confidence intervals (CIs), with the two-sided significance level set at 0.05. Statistical analyses were conducted in STATA 17.0 (StataCorp, College Station, TX).

## Results

Median follow up for our cohort of 14,976 women was 4.51 years (range 0.01–10.99 years), with 1,341 dying of breast cancer, and 884 dying from other causes. Of these 14,976 women, 27% were dispensed a statin after diagnosis (Table 1). Higher proportions of statin users compared to nonusers were diagnosed in earlier years of the study period, were older, were from more deprived areas, were treated in a public facility, and were more likely to have their cancer detected by screening. A higher proportion of statin users than nonusers were also more likely to have used other medications (beta blockers, ACEIs, ARBs, diuretics, metformin, and aromatase inhibitors, but not tamoxifen) and to have had documented comorbidities (any cardiac condition, diabetes, stroke, and renal disease) (*p* < 0.05 for all differences).

**Table 1:** Characteristics of breast cancer patients by statin use

Characteristics	Statin use after diagnosis	
	Ever-*n* (%)	Never-*n* (%)
Overall	4,060	10,916
Year of diagnosis		
2007–2008	677 (17)	1,250 (11)
2009–2010	832 (20)	1,824 (17)
2011–2012	886 (22)	2,383 (22)
2013–2014	846 (21)	2,670 (24)
2015–2016	819 (20)	2,789 (26)
Age at diagnosis		
< 50	375 (9)	3,881 (36)
50–59	899 (22)	3,099 (28)
60–69	1,501 (37)	2,326 (21)
70–79	836 (21)	942 (9)
80+	449 (11)	668 (6)
Ethnic group		
European	2,825 (70)	8.213 (75)
Maori	496 (12)	932 (9)
Pacific	332 (8)	616 (6)
Asian	305 (8)	910 (8)
Other	102 (3)	245 (2)
NZDep^a^		
1–2	585 (14)	2,054 (19)
3–4	692 (17)	2,229 (20)
5–6	844 (21)	2,160 (20)
7–8	666 (16)	1,591 (15)
9–10	816 (20)	1,398 (13)
Unknown	457 (11)	1,484 (14)
Urban/rural		
Urban	3,327 (82)	8,576 (79)
Rural	278 (7)	863 (8)
Unknown	455 (11)	1,477 (14)
Status of facility		
Public	3,005 (74)	6,922 (63)
Private	1,055 (26)	3,994 (37)
Register		
Auckland	2,371 (58)	5,885 (54)
Christchurch	525 (13)	1,899 (17)
Waikato	709 (17)	1,536 (14)
Wellington	455 (11)	1,596 (15)
Cancer stage		
1	1,956 (48)	4,790 (44)
2	1,360 (34)	3,764 (34)
3	473 (12)	1,457 (13)
4	121 (3)	575 (5)
Unknown	150 (4)	330 (3)
Cancer grade		
Well differentiated	968 (24)	2,305 (21)
Moderately differentiated	1,861 (46)	4,696 (43)
Poorly differentiated	1,0 2 (26)	3,252 (30)
Unknown	169 (4)	663 (6)
Method of diagnosis		
Symptomatic	2,194 (54)	6,574 (60)
Screen detected	1,866 (46)	4,342 (40)
Lymphovascular invasion		
No	2,559 (63)	6,386 (59)
Yes	1,275 (31)	3,798 (35)
Unknown	226 (6)	732 (7)
Molecular subtype		
HER2+ non-luminal	171 (4)	610 (6)
Luminal A	892 (22)	2,125 (19)
Luminal B HER2−	1,927 (47)	5,071 (46)
Luminal B HER2+	309 (8)	1,164 (11)
Triple negative	440 (11)	1,103 (10)
Unknown	321 (8)	843 (8)
ER status		
Positive	3,361 (83)	8,933 (82)
Negative	641 (16)	1,782 (16)
Unknown	58 (1)	201 (2)
Other medication use after diagnosis		
Beta blockers	1,667 (41)	1,528 (14)
ACEIs	2,164 (53)	1,964 (18)
ARBs	708 (17)	645 (6)
Diuretics	1,566 (39)	1,859 (17)
Metformin	1,013 (25)	404 (4)
Tamoxifen	1,383 (34)	5,122 (47)
Aromatase inhibitors	1,854 (46)	4,066 (37)
Hospitalised comorbidities^b^		
Any cardiac condition	785 (19)	494 (5)
Diabetes	424 (10)	141 (1)
Stroke	176 (4)	84 (1)
COPD	101 (2)	107 (1)
Peripheral vascular disease	53 (1)	17 (0.2)
Renal disease	107 (3)	54 (0.5)

The chi-square test was statistically significant (*p* < 0.05) for every variable except for ER status

^a^The NZDep is an area-based measure of socioeconomic deprivation in New Zealand. 1 represents the areas with the least deprived scores and 10 the areas with the most deprived scores

^b^Comorbidities included those in a patient’s hospital records five years before breast cancer diagnosis. Cardiac conditions included any of angina, arrhythmia, congestive heart failure, hypertension, myocardial infarction, ‘other cardiac conditions’, and valve disease

We compared the risk of BCD associated with statin use (vs non-use) after diagnosis (Table 2). In the unadjusted model, statin use was associated with a decreased risk of BCD (HR_crude_ = 0.88; 95% CI 0.77–1.00). This decreased risk was further reduced after adjustment for demographic and breast cancer clinical factors (HR = 0.81; 0.70–0.94), and reduced again with further adjustment for other medication use and comorbidities, with the fully adjusted model indicating a statistically significant 26% reduction in BCD associated with statin use vs non-use (HR = 0.74; 0.63–0.86). In exploratory analysis in which we additionally adjusted for chemotherapy and radiotherapy (both as binary yes/no variables and including both neoadjuvant and adjuvant therapies), the HR was not attenuated (HR = 0.74; 0.63–0.87, data not shown). Lagging statin use by various lengths of time did not substantially alter the HR. A similar finding was noted when adjusting for the competing risk of death from other causes (SHR = 0.73; 0.60–0.88). When adjusting for demographic variables, clinical variables, comorbidities, and other medication use in four steps, it was found that demographic variables were the strongest confounders of the association (Supplementary Table 1).

**Table 2:** Associations of breast cancer-specific survival with post-diagnostic use of statins (vs non-use) in breast cancer patients, by total dose

Medication usage after diagnosis	No. Breast cancer deaths	No. person-years	Unadjusted HR (95% CI)	Adjusted^a^ HR (95% CI)	Fully adjusted^b^ HR (95% CI)
Statin nonuser	1,068	55,609	1.00	1.00	1.00
Statin user	273	17,179	0.88 (0.77–1.00)	0.81 (0.70–0.94)	0.74 (0.63–0.86)
1–90 DDDs (0–3 months)	44	1,816	1.32 (0.98–1.79)	1.14 (0.84–1.56)	1.03 (0.75–1.40)
91–181 DDDs (3–6 months)	34	1,800	1.04 (0.74–1.47)	1.00 (0.71–1.42)	0.88 (0.62–1.25)
182–272 DDDs (6–9 months)	16	1,215	0.68 (0.41–1.11)	0.66 (0.40–1.08)	0.56 (0.34–0.93)
273–364 DDDs (9 months–1 year)	19	1,262	0.74 (0.47–1.17)	0.57 (0.36–0.90)	0.46 (0.29–0.74)
365–729 DDDs (1–2 years)	68	3,481	0.97 (0.76–1.24)	0.88 (0.69–1.14)	0.83 (0.64–1.08)
730–1094 DDDs (2–3 years)	26	2,235	0.61 (0.41–0.90)	0.62 (0.42–0.92)	0.59 (0.40–0.89)
1095 or more DDDs (3 or more years)	66	5,370	0.78 (0.60–1.00)	0.74 (0.57–0.97)	0.68 (0.51–0.89)
Statin user, 6-month lag	241	15,189	0.87 (0.76–1.01)	0.81 (0.69–0.94)	0.77 (0.65–0.91)
Statin user, 1-year lag	202	13,288	0.84 (0.72–0.98)	0.77 (0.65–0.91)	0.72 (0.60–0.86)
Statin user, 2-year lag	140	9,933	0.85 (0.71–1.03)	0.78 (0.64–0.95)	0.76 (0.61–0.94)
Statin user, adjusting for competing risks	273	17,180	0.86 (0.75–0.98)	0.81 (0.69–0.96)	0.73 (0.60–0.88)

^a^First adjustment controlled for date of dx, age, ethnic group, deprivation, urban/rural status, public/private status of the facility, register, stage, grade, mode of detection, lymphovascular invasion, and receptor status

^b^Second adjustment controlled for the previous covariates as well as other drug use and hospitalised comorbidities (other drugs including beta blockers, ACEIs, ARBs, diuretics, metformin, tamoxifen, and aromatase inhibitors. Comorbidities including any cardiac condition as yes/no, diabetes, stroke, COPD, peripheral vascular disease, and renal disease). Other drug covariates were modelled in the same fashion as statins (except for dose analysis, in which other drugs were classified as user/nonuser and modelled as time varying covariates)

^c^DDDs refer to daily defined doses

^d^The *p* value for linear trend for the fully adjusted dose analysis was 0.0012

In the dose analysis, the highest HR was observed during the initial 0–3 months of statin use (HR = 1.03; 0.75–1.40, Table 2). The risk of BCD generally decreased with increasing duration of use (although the results were inconsistent with the HR both decreasing and increasing over time), and a 32% reduction in BCD was found in those who took a statin for the equivalent of three or more years (HR = 0.68; 0.51–0.89). Excluding nonusers (i.e. among statin users only), the overall p value for linear trend was 0.0012.

In subgroup analyses (Table 3), a statistically significant protective effect on BCD was found in women who were ER+ (HR = 0.77; 0.63–0.94), but there was no association found in ER- patients (HR = 1.00; 0.76–1.31). When cancers were divided into triple negative and non-triple negative tumours, no association was found in either group. A statistically significant protective effect was found in postmenopausal women (HR = 0.74; 0.63–0.88), whereas there was no association in premenopausal women (HR = 0.98; 0.59–1.62). A statistically significant protective effect was found in women with advanced stage disease (HR = 0.65; 0.49–0.84), while there was only a suggestion of a protective effect in those with early-stage disease (HR = 0.89; 0.71–1.11). Lastly, there was a more protective effect found in prevalent users of statins (HR = 0.69; 0.58–0.83) than in ‘new’/incident users of statins (HR = 0.91; 0.69–1.19).

**Table 3:** Associations of breast cancer-specific survival with post-diagnostic use of statins (vs non-use) in breast cancer patients, subgroup analyses

Medication usage after diagnosis	No. breast cancer deaths	No. person-years	Unadjusted HR (95% CI)	Adjusted^a^ HR (95% CI)	Fully adjusted^b^ HR (95% CI)
Statin nonuser	654	46,183	1.00	1.00	1.00
Statin user, ER positive	165	14,316	0.83 (0.69–0.98)	0.83 (0.69–1.00)	0.77 (0.63–0.94)
Statin nonuser	340	8,777	1.00	1.00	1.00
Statin user, ER negative^c^	100	2,617	1.08 (0.86–1.35)	0.99 (0.77–1.27)	1.00 (0.76–1.31)
Statin nonuser	218	5,391	1.00	1.00	1.00
Statin user, triple negative	71	1,775	1.06 (0.81–1.39)	0.89 (0.65–1.22)	0.91 (0.65–1.28)
Statin nonuser	561	46,897	1.00	1.00	1.00
Statin user, not triple negative^d^	143	14,170	0.82 (0.68–0.98)	0.95 (0.78–1.16)	0.86 (0.70–1.07)
Statin nonuser	386	22,324	1.00	1.00	1.00
Statin user, pre-menopausal	25	1,763	0.83 (0.55–1.24)	1.19 (0.78–1.81)	0.98 (0.59–1.62)
Statin nonuser	678	33,066	1.00	1.00	1.00
Statin user, post-menopausal	247	15,388	0.86 (0.74–1.00)	0.80 (0.68–0.93)	0.74 (0.63–0.88)
Statin nonuser,	445	50,477	1.00	1.00	1.00
Statin user, early stage^e^	152	15,708	1.05 (0.87–1.26)	0.98 (0.80–1.20)	0.89 (0.71–1.11)
Statin nonuser,	523	3,834	1.00	1.00	1.00
Statin user, advanced stage^f^	92	915	0.81 (0.65–1.01)	0.67 (0.53–0.86)	0.65 (0.49–0.84)
Statin user, ‘new’ user^g^	60	4,399	0.85 (0.65–1.11)	0.93 (0.71–1.21)	0.91 (0.69–1.19)
Statin user, ‘prevalent’ user^h^	213	12,780	0.88 (0.76–1.02)	0.78 (0.67–0.92)	0.69 (0.58–0.83)

^a^First adjustment controlled for date of dx, age, ethnic group, deprivation, urban/rural status, public/private status of the facility, register, stage, grade, mode of detection, lymphovascular invasion, and receptor status

^b^Second adjustment controlled for the previous covariates as well as other drug use and hospitalised comorbidities (other drugs including beta blockers, ACEIs, ARBs, diuretics, metformin, tamoxifen, and aromatase inhibitors. Comorbidities including any cardiac condition as yes/no, diabetes, stroke, COPD, peripheral vascular disease, and renal disease). Other drug covariates were modelled in the same fashion as statins

^c^Those with a missing ER status were not included in this analysis

^d^Those with a missing molecular subtype were not included in this analysis

^e^Restricted to patients with stage 1, stage 2, or stage 3a cancers. Patients with an ‘unknown’ stage were excluded in this analysis

^f^Restricted to patients with stage 3b, stage 3c, or stage 4 cancers. Patients with an ‘unknown’ stage were excluded in this analysis

^g^A ‘new’ statin user was defined as a woman who did not have a statin dispensing in the year prior to her breast cancer diagnosis

^h^A ‘prevalent’ statin user was defined as a woman who did have a statin dispensing in the year prior to her breast cancer diagnosis

When comparing statin users to a statin nonuser group who used aspirin (Table 4), a 28% decreased risk of BCD was found (HR = 0.72; 0.59–0.88). This decreased risk was not altered by lagging statin use by various lengths of time.

**Table 4:** Associations of breast cancer-specific survival with post-diagnostic use of statins (vs non-use) in breast cancer patients, using a comparison group of nonusers who were dispensed aspirin

Medication usage after diagnosis	No. breast cancer deaths	No. person-years	Unadjusted HR (95% CI)	Adjusted^a^ HR (95% CI)	Fully adjusted^b^ HR (95% CI)
Statin nonusers who used aspirin	178	5,973	1.00	1.00	1.00
Statin user	273	17,179	0.52 (0.43–0.63)	0.71 (0.59–0.87)	0.72 (0.59–0.88)
Statin user, 6-month lag	241	15,189	0.60 (0.50–0.73)	0.78 (0.63–0.95)	0.76 (0.62–0.95)
Statin user, 1-year lag	202	13,288	0.62 (0.50–0.76)	0.78 (0.62–0.96)	0.74 (0.59–0.93)
Statin user, 2-year lag	140	9,933	0.68 (0.53–0.86)	0.80 (0.63–1.03)	0.74 (0.57–0.96)

^a^First adjustment controlled for date of dx, age, ethnic group, deprivation, urban/rural status, public/private status of the facility, register, stage, grade, mode of detection, lymphovascular invasion, and receptor status

^b^Second adjustment controlled for the previous covariates as well as other drug use and hospitalised comorbidities (other drugs including beta blockers, ACEIs, ARBs, diuretics, metformin, tamoxifen, and aromatase inhibitors. Comorbidities including any cardiac condition as yes/no, diabetes, stroke, COPD, peripheral vascular disease, and renal disease). Other drug covariates were modelled in the same fashion as statins

In the analysis considering recurrence as the outcome (Table 5), there was no statistically significant association found between statin use and BCR (HR = 0.94; 0.80–1.11). There was also no statistically significant association found between statin use and local recurrence (HR = 0.95; 0.73–1.25) or distant recurrence (HR = 0.96; 0.80–1.15).

**Table 5:** Associations of breast cancer recurrence with post-diagnostic use of statins (vs non-use) in breast cancer patients

Medication usage after diagnosis	No. breast cancer recurrences	No. person-years	Unadjusted HR (95% CI)	Adjusted^a^ HR (95% CI)	Fully adjusted^b^ HR (95% CI)
Statin nonuser	943	43,270	1.00	1.00	1.00
Statin user	273	13,698	0.90 (0.79–1.03)	0.93 (0.80–1.08)	0.94 (0.80–1.11)
Local recurrence					
Statin nonuser	377	44,079	1.00	1.00	1.00
Statin user	98	13,923	0.80 (0.64–1.00)	0.88 (0.69–1.12)	0.95 (0.73–1.25)
Distant recurrence					
Statin nonuser	727	43,922	1.00	1.00	1.00
Statin user	218	13,918	0.94 (0.81–1.09)	0.94 (0.80–1.11)	0.96 (0.80–1.15)

^a^First adjustment controlled for date of dx, age, ethnic group, deprivation, urban/rural status, public/private status of the facility, register, stage, grade, mode of detection, lymphovascular invasion, and receptor status

^b^Second adjustment controlled for the previous covariates as well as other drug use and hospitalised comorbidities (other drugs including beta blockers, ACEIs, ARBs, diuretics, metformin, tamoxifen, and aromatase inhibitors. Comorbidities including any cardiac condition as yes/no, diabetes, stroke, COPD, peripheral vascular disease, and renal disease). Other drug covariates were modelled in the same fashion as statins (except for dose analysis, in which other drugs were classified as user/nonuser and modelled as time varying covariates)

^c^Restricted to patients with stage 1, stage 2, or stage 3a cancers. Patients with an ‘unknown’ stage were excluded in this analysis

In the analysis with all-cause mortality as the outcome (Supplementary Table 2), statin use was associated with a statistically significant decreased risk of dying from any cause (HR = 0.86; 0.77–0.96).

## Discussion

There was a statistically significant decreased risk between any statin use after breast cancer diagnosis and BCD in this large New Zealand population-based cohort study of patients with breast cancer after adjustment for demographic and clinical factors, comorbidities, and other medication use. There were suggestions of effect modification across subgroups, in that statins were more protective for ER+ cancers, in postmenopausal women, in late-stage patients, as well as in ‘prevalent’ statin users.

Our primary finding is consistent with a number of previous studies indicating a statistically significant protective effect between statin use and BCD in their fully adjusted analyses [16–22]. For example, a large Swedish study conducted on 20,559 breast cancer patients found regular statin use to be associated with a 23% reduction in BCD (HR = 0.77; 0.63–0.94) [16], a very similar result to our primary finding. Further, Nielsen and colleagues found regular statin use to be associated with a 12% reduction in BCD in a large cohort of 46,562 Danish breast cancer patients [21]. Several other observational studies have found statins to have no statistically significant effect on BCD [23–32]. For example, null associations have been found in a Scottish study of 15,140 breast cancer patients (HR = 0.95; 0.79–1.15) [24] and in an Irish study of 4,243 breast cancer patients (HR = 0.88; 0.66–1.17) [25].

In the dosing analysis (Table 2), the risk generally (although inconsistently) decreased as the dosage increased over time (p for trend = 0.0012) (Table 2). One study found a suggestion of statins becoming more protective with increasing doses [17], however a number have found no evidence of a dose–response relationship [16, 18, 23, 24]. An absence of a dose–response relationship may be what would be expected, as some of the small clinical trials that have been conducted on statins and breast cancer outcomes thus far have indicated that any potential protective effect of statins may be exhibited even when taken for very short periods of time (e.g. 2–6 weeks) [43, 44]. As evidenced by Supplementary Table 3, those who took statins in the lowest dose categories generally had a shorter median time to death/last follow up than those with took statins in the higher dose categories (a similar phenomenon was evidenced in a previous paper of ours for beta blockers [81]). Therefore, the slightly higher HRs observed in the 0–3 month and 3–6 month dose categories may in fact be an artefact of women in these dose categories being dispensed statins towards the end of their life. Indeed, when removing the lowest two dose categories from the same analysis, the p for trend increased from 0.0012 to 0.3263 (data not shown).

Statins were more protective in ER+ cancers (HR = 0.77; 0.63–0.94) than in ER- cancers (HR = 1.00; 0.76–1.31) (Table 3). This finding is in agreement with one other study that found a statistically significant protective effect for BCD in ER+ cancers but no effect in ER- cancers [17], however three other studies that set out to examine effect modification by ER status found no evidence of a different effect between groups [24–26]. The more protective effect in ER+ tumours is thought to result from statins lowering levels of cholesterol metabolite 27-hydroxycholesterol (27HC), a selective estrogen receptor modulator that can regulate ER-dependent tumour growth [46, 47, 82]. It may be that the lowering of this metabolite only has an effect in postmenopausal women (because the estrogen receptors are already fully stimulated by high circulating estrogen and/or they are blocked by taxomifen in premenopausal women) and/or statins reduce estrogen sourced from extraovarian subcutaneous adipose tissue in postmenopausal women combined with aromatase inhibitors being used more often in postmenopausal women (which also lower estrogen) [83, 84], which would explain our finding of a protective effect of statins in postmenopausal women but no effect in premenopausal women. Only one other observational study has carried out a sensitivity analysis restricted to postmenopausal women only, and their results are very similar to ours [29]. No differential effect was found in triple negative vs non-triple negative tumours, which contrasts with a previous study conducted in the USA that found a statistically significant protective effect in triple negative tumours only [20].

We found a statistically significant protective effect in advanced stage (3b, 3c, and 4) patients (HR = 0.65; 0.49–0.84), but no effect in early stage (1, 2, and 3a) patients (HR = 0.89; 0.71–1.11) (Table 3). To our knowledge, this is the first study to show a differential effect of statins by stage. One other study stratified by stage and did not find any evidence of effect modification [24]. It may be hypothesised that the slightly higher HR in early-stage patients in our study is due to prescribing bias by stage/prognosis, in that we might expect a lot of very ill patients to be removed from the statin nonuser group in this analysis relative to including all patients. However, other analyses that examined prescribing bias by stage/prognosis, including splitting up follow up time into less than three years and three or more years, as well considering pre diagnostic statin use as the exposure of interest did not show substantially different results relative to our primary analysis (Supplementary Tables 4 and 5). Similarly, one other study also found very similar findings to their main analysis when considering pre diagnostic statin use as the exposure [18]. The more protective effect in late-stage patients points towards statins exerting their effect on late-stage tumour progression/metastasis, a finding that is consistent with recent experimental and observational studies [13, 36, 85, 86]. While we did not find a protective effect on distant metastasis/recurrence (nor on local recurrence or all recurrences combined, Table 5), this lack of an effect may be attributable to our relatively poor data on recurrence rather than the absence of a true association (i.e. our data only recorded recurrences when women presented to the breast cancer clinic, and there were no routine follow ups to ascertain women’s recurrence status).

In the analysis in which users were split up into ‘new’ and ‘prevalent’ users, a more protective effect was found in ‘prevalent’ users (HR = 0.69; 0.58–0.83) than in ‘new’ users (HR = 0.91; 0.69–1.19). This result is in line with what we would expect, in that prevalent users are likely to have tolerated the medication well and are also likely to have a higher propensity for health seeking behaviours (in general) than statin initiators [77]. Although we were able to adjust for a number of covariates that indicate the ‘healthiness’ of women, there is likely some residual confounding remaining that we were unable to capture. Therefore, ‘prevalent’ users (which made up 70% of our statin users and 78% of breast cancer deaths in statin users) are likely to have a spurious survival advantage over and above any potential causal effect. Further, it is conceivable that some of our covariates measured at baseline would be affected by statin use prior to breast cancer diagnosis, perhaps inducing some overadjustment bias [87]. It is worthy to note that the ‘new’ user group had a lower number of events (and follow up time) than ‘prevalent’ users. As such, the failure of our study to find an effect in this group may be indicative of a lack of power rather than the absence of a true effect. Indeed, a Finnish study enrolling 31,236 breast cancer patients found the protective effect of statins to hold up in ‘new’ users [18], while Cardwell and colleagues found an even more protective effect in ‘new’ users than in ‘prevalent’ users [23]. Finally, it is also worth noting that ‘new’ statin users were much more likely to be short-term users than ‘prevalent’ statin users (Supplementary Table 6). As such, the slightly higher HR in ‘new’ users may also be a function of this group being more likely to use statins towards the end of their life than ‘prevalent’ users.

The primary strength of our study is that we had a large cohort of patients with breast cancer followed up over a relatively long time period sourced from four population-based databases. The databases have been checked against the National Cancer Registry and found to be at least 99% complete, and the registry data we used contains more comprehensive and accurate information than the national data sources [88–90]. Our pharmaceutical data was derived from a high quality and automated national database, and there was no recall bias [91] associated with medication records as a result. Furthermore, unlike many other countries, New Zealand records medication dispensings instead of prescriptions, which are a stronger proxy for medication adherence. We also conceptualised medication use as time varying covariates, and therefore avoided the introduction of immortal time bias that invariably biases results in favour of the medication [70].

Our study also has limitations. We did not have access to primary care data, which meant that our comorbidity data was restricted to hospital admissions in the relevant timeframe. Furthermore, this limited access to a range of potential confounders such as body mass index, alcohol intake, and smoking status, all of which would generally be available through general practitioner records. However, these limitations in residual confounders were somewhat mitigated by the use of a more balanced comparison group. Serum cholesterol levels were also not available in our data, which would likely induce confounding by indication as cholesterol has been associated with both the risk and prognosis of breast cancer [40]. Over ninety nine percent of breast cancer deaths in our study were in lipophilic statin users, and we therefore did not have the power to explore the relationship between different statin types (lipophilic vs hydrophilic) and breast cancer outcomes. However, preclinical studies have consistently indicated that lipophilic statins are the only statins to have anti proliferative effects on breast cancer cells [14, 92, 93]. Finally, we also did not have the power to examine the association between other types of cholesterol lowering medication and breast cancer outcomes due to their infrequent use among women in our cohort.

In conclusion, we found a statistically significant protective effect between post-diagnostic statin use and BCD in this large population-based study on NZ patients with breast cancer. The protective effect of statins was attenuated when considering ‘new’ users as the exposure, but there was a more protective effect found in ER+ patients, postmenopausal women, and in women with advanced stage disease. Further research is warranted in these subgroups to ascertain a targeted population of breast cancer patients that may benefit from statin therapy in the adjuvant setting.

## Supplementary Information

Below is the link to the electronic supplementary material.

Supplementary file1 (DOCX 24 KB)

## Data Availability

The datasets used in this study contain personal information and are not publicly available, but may be requested from the Breast Cancer Foundation New Zealand and the Ministry of Health (NZ).
