# Supplementary material for: Post-diagnostic statin use and breast cancer-specific mortality: a population-based cohort study
Source: Breast Cancer Res Treat. 2023 Mar 17;199(1):195–206. doi: 10.1007/s10549-022-06815-w (PMC10147735; doi:10.1007/s10549-022-06815-w)
Supplement: Supplementary file 1 — Supplementary file1 (DOCX 24 KB) [file 10549_2022_6815_MOESM1_ESM.docx]

**Supplementary tables**

**Supplementary Table 1. Associations of breast cancer specific survival with post-diagnostic use of statins (vs non-use) in breast cancer patients.**

| **Medication Usage After Diagnosis** | **No. Breast cancer deaths** | **No. person-years** | **Unadjusted HR (95% CI)** | **Adjusted^a^ HR (95% CI)** | **Adjusted^b^ HR (95% CI)** | **Adjusted^c^ HR (95% CI)** | **Fully adjusted^d^ HR (95% CI)** |
| --- | --- | --- | --- | --- | --- | --- | --- |
| Statin nonuser | 1,068 | 55,609 | 1.00 | 1.00 | 1.00 | 1.00 | 1.00 |
| Statin user | 273 | 17,179 | 0.88 (0.77-1.00) | 0.72 (0.62-0.82) | 0.81 (0.70-0.94) | 0.77 (0.66-0.89) | 0.74 (0.63-0.86) |

*^a^First adjustment controlled for* *date of dx, age, ethnic group, deprivation, urban/rural status, public/private status of the facility, and register.*

*^b^Second adjustment controlled for the previous covariates as well as* *stage, grade, mode of detection, lymphovascular invasion, and receptor status.*

*^c^Third adjustment controlled for the previous covariates as well hospitalised comorbidities. Comorbidities included any cardiac condition as yes/no, diabetes, stroke, COPD, peripheral vascular disease, and renal disease.*

*^d^Fourth adjustment controlled for the previous covariates as well as other drug use including beta blockers, ACEIs, ARBs, diuretics, metformin, tamoxifen, and aromatase inhibitors. Other drug covariates were modelled in the same fashion as statins.*

**Supplementary Table 2. Associations of all-cause mortality with post-diagnostic use of statins (vs non-use) in breast cancer patients.**

| **Medication Usage After Diagnosis** | **No. deaths** | **No. person-years** | **Unadjusted HR (95% CI)** | **Adjusted**^a^ **HR (95% CI)** | **Fully adjusted**^b^ **HR (95% CI)** |
| --- | --- | --- | --- | --- | --- |
| Statin nonuser | 1,567 | 55,609 | 1.00 | 1.00 | 1.00 |
| Statin user | 658 | 17,179 | 1.37 (1.25-1.50) | 1.03 (0.93-1.13) | 0.86 (0.77-0.96) |

*^a^First adjustment controlled for date of dx, age, ethnic group, deprivation, urban/rural status, public/private status of the facility, register, stage, grade, mode of detection, lymphovascular invasion, and receptor status.*

*^b^Second adjustment controlled for the previous covariates as well as other drug use and hospitalised comorbidities (other drugs including beta blockers, ACEIs, ARBs, diuretics, metformin, tamoxifen, and aromatase inhibitors. Comorbidities including any cardiac condition as yes/no, diabetes, stroke, COPD, peripheral vascular disease, and renal disease). Other drug covariates were modelled in the same fashion as statins.*

**Supplementary Table 3. Median time to death/last follow up from women’s first statin dispensing, by dose category.**

| Dose category | Number of users who died due to breast cancer | Median time to death (in years) from their first statin dispensing | Number of users who died due to any cause | Median time to death (in years) from their first statin dispensing | Number of users who didn’t die | Median time to last follow up (in years) from their first statin dispensing |
| --- | --- | --- | --- | --- | --- | --- |
| 1-90 DDDs (0-3 months) | 44 | 0.70 | 84 | 0.81 | 241 | 1.89 |
| 91-181 DDDs (3-6 months) | 34 | 0.99 | 67 | 0.97 | 202 | 1.83 |
| 182-272 DDDs (6-9 months) | 16 | 0.88 | 36 | 1.84 | 153 | 1.51 |
| 273-364 DDDs (9 months-1 year) | 19 | 1.13 | 46 | 1.15 | 162 | 1.78 |
| 365-729 DDDs (1 year-2 years) | 68 | 2.09 | 151 | 2.24 | 564 | 2.54 |
| 730-1094 DDDs (2 years-3 years) | 26 | 2.74 | 70 | 3.30 | 476 | 3.67 |
| 1095 or more DDDs (3 or more years) | 66 | 3.95 | 204 | 4.74 | 1,604 | 5.85 |

**Supplementary Table 4. Associations of breast cancer specific survival with post-diagnostic use of statins (vs non-use) in breast cancer patients.**

| **Medication Usage After Diagnosis** | **No. Breast cancer deaths** | **No. Person-years** | **Unadjusted HR (95% CI)** | **Adjusted**^a^ **HR (95% CI)** | **Fully adjusted**^b^ **HR (95% CI)** |
| --- | --- | --- | --- | --- | --- |
| **Less than three years of follow up time** |  |  |  |  |  |
| Statin nonuser | 670 | 31,735 | 1.00 | 1.00 | 1.00 |
| Statin user | 160 | 7,925 | 0.96 (0.81-1.15) | 0.83 (0.69-1.00) | 0.78 (0.64-0.96) |
|  |  |  |  |  |  |
| **Three or more years of follow up time** |  |  |  |  |  |
| Statin nonuser | 398 | 23,873 | 1.00 | 1.00 | 1.00 |
| Statin user | 113 | 9,254 | 0.77 (0.62-0.95) | 0.84 (0.66-1.05) | 0.72 (0.55-0.92) |

*^a^First adjustment controlled for date of dx, age, ethnic group, deprivation, urban/rural status, public/private status of the facility, register, stage, grade, mode of detection, lymphovascular invasion, and receptor status.*

*^b^Second adjustment controlled for the previous covariates as well as other drug use and hospitalised comorbidities (other drugs including beta blockers, ACEIs, ARBs, diuretics, metformin, tamoxifen, and aromatase inhibitors. Comorbidities including any cardiac condition as yes/no, diabetes, stroke, COPD, peripheral vascular disease, and renal disease). Other drug covariates were modelled in the same fashion as statins.*

**Supplementary Table 5. Associations of breast cancer specific survival with pre-diagnostic use of statins (vs non-use) in breast cancer patients.**

| **Medication Usage in the Year Before Diagnosis** | **No. Breast cancer deaths** | **No. Person-years** | **Unadjusted HR (95% CI)** | **Adjusted**^a^ **HR (95% CI)** | **Fully adjusted**^b^ **HR (95% CI)** |
| --- | --- | --- | --- | --- | --- |
| Statin nonuser | 1090 | 58,781 | 1.00 | 1.00 | 1.00 |
| Statin user^c^ | 251 | 14,007 | 0.96 (0.83-1.10) | 0.78 (0.67-0.90) | 0.72 (0.61-0.85) |

*^a^First adjustment controlled for date of dx, age, ethnic group, deprivation, urban/rural status, public/private status of the facility, register, and receptor status.*

*^b^Second adjustment controlled for the previous covariates as well as other drug use and hospitalised comorbidities (other drugs including beta blockers, ACEIs, ARBs, diuretics, metformin, tamoxifen, and aromatase inhibitors. Comorbidities including any cardiac condition as yes/no, diabetes, stroke, COPD, peripheral vascular disease, and renal disease). Other drug covariates were modelled in the same fashion as statins.*

*^c^Statin users were defined as those with any dispensing of a statin in the year prior to breast cancer diagnosis.*

**Supplementary Table 6. Proportion of ‘new’ users, proportion of ‘prevalent’ users, and proportion of users who died due to breast cancer, by dose category.**

| Dose category | Percentage of ‘new’ users^a^ | Percentage of ‘prevalent’ users^b^ | Percentage of users who died due to breast cancer |
| --- | --- | --- | --- |
| 1-90 DDDs (0-3 months) | 71.08 | 28.92 | 13.54 |
| 91-181 DDDs (3-6 months) | 56.13 | 43.87 | 12.64 |
| 182-272 DDDs (6-9 months) | 41.27 | 58.73 | 8.47 |
| 273-364 DDDs (9 months-1 year) | 35.58 | 64.42 | 9.13 |
| 365-729 DDDs (1 year-2 years) | 26.99 | 73.01 | 9.51 |
| 730-1094 DDDs (2 years-3 years) | 27.11 | 72.89 | 4.76 |
| 1095 or more DDDs (3 or more years) | 19.91 | 80.09 | 3.65 |

*^a^A ‘new’ user was defined as women who did not have a statin dispensing in the year prior to their breast cancer diagnosis.*

*^b^A ‘prevalent’ user was defined as women who did have a statin dispensing in the year prior to their breast cancer diagnosis.*
